# Evaluation of Concentration Polarization Due to the Effect of Feed Water Temperature Change on Reverse Osmosis Membranes

**DOI:** 10.3390/membranes13010003

**Published:** 2022-12-21

**Authors:** Germán Eduardo Dévora-Isiordia, Cristian Ascención Cásares-De la Torre, Deemi Paola Morales-Mendívil, Rosario Montoya-Pizeno, Nicolás Velázquez-Limón, Jesús Armando Aguilar-Jiménez, Juan Ríos-Arriola

**Affiliations:** 1 Departamento de Ciencias del Agua y Medio Ambiente, Instituto Tecnológico de Sonora, Calle 5 de Febrero 818 Sur, Ciudad Obregón 85000, Mexico; 2 Centro de Estudio de las Energías Renovables, Instituto de Ingeniería, Universidad Autónoma de Baja California, Mexicali 21280, Mexico; 3 Departamento de Ingeniería Química, Instituto Tecnológico de Sonora, Calle 5 de Febrero 818 Sur, Ciudad Obregón 85000, Mexico; 4 Facultad de Ingeniería, Universidad Autónoma de Baja California, Mexicali 21280, Mexico

**Keywords:** desalination, concentration polarization, reverse osmosis, water scarcity

## Abstract

Water is a necessary resource for life development. Its excessive consumption has a negative impact, generating scarcity problems worldwide. Desalination is an alternative to solve these problems; its objective is to reduce the concentration of total dissolved solids to levels suitable for consumption. The most widely used desalination technology is reverse osmosis, which works by means of semipermeable membranes; however, lack of knowledge or wrong operation cause phenomena such as concentration polarization, which reduces the effective area for mass transfer in the membrane, increasing the energy consumption of the process. The objective of the present study is to evaluate the concentration polarization (β) of the concentration in reverse osmosis membranes by varying the temperature in the feed water (23, 25.5, 28, and 35 °C) for different concentrations (5000 and 10,000 mg L^−1^) in order to reduce its impact on energy consumption (kWh m^−3^). The results show that as the temperature increases, the specific energy consumption decreases for both concentrations. In the 5000 mg L^−1^ tests, the specific energy consumption decreased by 0.590 kWh m^−3^, representing 12.5%. For 10,000 mg L^−1^ tests, the specific energy consumption shows a reduction of 0.72 kWh m^−3^, which represents a percentage decrease of 14.54%.

## 1. Introduction

The accelerated population growth and industrialization present during the last decades around the world have significantly increased the demand for resources; therefore, it is becoming more difficult to meet the basic needs of human beings, limiting the availability of drinking water, food, and electrical energy [1]. Although water covers most of the Earth, only about 2.5% of it is available as fresh water. However, the percentage of fresh water available for consumption is much lower because nearly three-quarters of this resource is frozen in glaciers and ice caps, making its use in the short term practically impossible [2,3].

It is estimated that by 2030 there will be a 40% water deficit worldwide [4]. For example, Mexico currently has 94% of its rivers and lakes polluted, dozens of cities with insufficient drinking water, and 50% of its water wasted; this country has been going through a critical water shortage situation for several years, and it has only been increasing [5,6]. About 77% of the national population, located in the arid and semi-arid states of the northern region of the country, are the most affected in terms of lack and total scarcity of water.

Desalination technologies appear to be an attractive solution to satisfy part of the global water shortage. Desalination consists of taking water with a high concentration of total dissolved solids (TDS) and passing it through a treatment process until it reaches the appropriate concentration ranges for its respective use [7]. In order to obtain fresh water through desalination, two types of systems can be used, which are categorized as: thermal and membrane systems [8]. The latter system separates the feed water into two streams, one of water with low concentration called -permeate- and another with the concentrate of the rejected salts called -*rejection*- [9]. Within this category of membrane desalination systems, the most used technology is reverse osmosis (RO).

**Figure 1:**
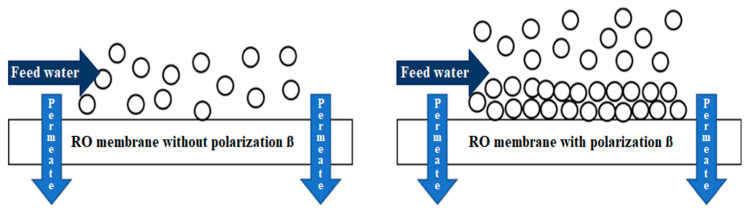
Concentration polarization in reverse osmosis membranes.

However, certain factors require special attention during the RO process. One of these factors is the so-called -*concentration polarization factor*-, which causes a series of phenomena that affect RO membranes directly and, consequently, the quality of the product water and the energy consumption of the process [10]. The concentration polarization occurs when, as feed water flows through the RO system, salts are retained by the semipermeable membrane and form a concentrated layer on its surface, as shown in Figure 1. The concentration at this point gradually increases because as the water flow continues to pass through the membranes, salts continue to accumulate, causing an even higher concentration than the inlet concentration -*scaling-* [11]. This phenomenon limits mass transfer in the process, causing an increase in; operating pressure, energy consumption, and scaling formation. It also decreases the quality and flux of the permeate [12].

McGovern and Lienhard [13] conducted a study with the aim of providing an explanation for the flux asymptote that arises due to concentration polarization when employing ultra-permeable reverse osmosis membranes used to desalinate seawater. The authors report a series of analytical formulas that clarify this limit for both single-stage and batch processes. These asymptotes illustrate that, although improvements in flux are not linear as permeability increases, the limit imposed on the mean flux by transport is approximately four times the current mean flux for seawater desalination and approximately twenty times the current mean flux for brackish water desalination.

Some correlations, new models, and/or the improvement of existing ones have been proposed in order to evaluate the behavior of concentration polarization in reverse osmosis membranes. Rathore et al. [14] developed a transport model that focuses on evaluating the formation of the concentration polarization layer and its behavior in reverse osmosis membranes. Their results show that as filtration increases, the amount of rejected solutes gradually increases; this is reflected in an increased thickness of the concentration polarization layer and a higher rate of mass transfer by counter diffusion in the boundary layer, the latter causing an accumulation of backward transported solutes resulting in higher accumulated concentrations along the boundary layer. Al-Mutaz and Alsubaie [15] combined the solution diffusion transport model and film theory to obtain an explicit expression for water flux through reverse osmosis membranes, and the authors mention that the formula will be helpful for the evaluation of the concentration polarization using parameters such as; permeability coefficients of water and solute, operating conditions and mass transfer coefficient. Their model was compared with data reported in the literature and showed a good agreement. Gu et al. [16] presented new dimensionless correlations for the concentration polarization and the friction factor obtained by analysis of computational fluid dynamics simulation data. Their results obtained with the new correlations are compared with those of the existing correlations in the literature; the authors report that there is good consistency in the predicted concentration polarization, with mean discrepancies of less than 6%.

Baghdadi et al. [17] theoretically investigated by means of FilmTec’s software the effect of temperature on the thickness of the concentration polarization layer in two types of membranes, with ex situ macromolecules and in situ macromolecules. The authors report that the permeate flux does not vary significantly as the temperature increases. However, the concentration polarization layer thickness decreases as the temperature increases and to a greater extent when the macromolecules are embedded in the membrane (in situ), while this decrease was less significant when the macromolecules act as a separate layer on the membrane surface (ex situ).

Li et al. [18] studied the performance of the miniature form of a scaled-up nanoporous membrane centrifuge for reverse-osmosis desalination. They studied the critical angular velocity for reverse osmosis desalination, concentration polarization, and energy efficiency against different design parameters; pore size, porous CNT centrifuge length, and freshwater flux rate. The authors report that the ion concentration density is almost zero at the membrane wall, implying that concentration polarization may not exist for their proposed nanoporous membrane centrifuge.

Zhang et al. [19] discovered a previously unknown mechanism that breaks the permeability–selectivity trade-off in using a rotating nanoporous graphene membrane with pores of two to four nanometers in diameter. Their results show that the rotating membrane exhibits almost 100% salt rejection even when the pore size is larger than that of hydrated ions. The authors report that no concentration polarization is observed from the radial concentration distribution and radial density distributions of water molecules and ions obtained from the molecular dynamics simulations.

Some recent work has focused on reducing concentration polarization with different types and configurations of spacers in the feed channel of reverse osmosis membranes. Ruiz-García and Pestana [20] simulated the effect of different spacer geometries in the feed channel for reverse osmosis membranes used to desalinate brackish water with different coefficients of permeability. Their results show that the geometry of the spacers has a greater impact on membranes with a higher coefficient of permeability. Wei et al. [21] simulated concentration polarization behavior in spirally wound reverse osmosis membranes. The authors report that for a concentration of 10,000 mg L^−1^ of salt and a pressure of 10.91 bar in the feed water, the amount of permeate went from 27.6 L/m^2^h to 24.1 L/m^2^h, as a result of concentration polarization in the absence of a spacer. On the contrary, the presence of the spacer decreased the concentration polarization, which increased the flow at the outlet up to 26.5 L/m^2^h. Sitaraman and Battiato [22] reported that with a non-uniform spacer configuration, substantial savings in pressure drop (∼40%), minor variations (∼2%) in concentration polarization are obtained, product water quality (∼1%), and water recovery (∼7%) compared to a uniform spacer configuration.

To summarize, it is clear that the study of polarization concentration reduction is a topic that is currently being addressed in different ways; therefore, models and simulations have been developed to understand its effects on the performance of RO systems and how to minimize them. Although it has been reported that some recently proposed novel membranes do not face this problem, it is still of interest to develop techniques to minimize it in RO systems that currently operate with conventional membranes, which are approximately 17,000 plants, equivalent to 81% of the total existing plants [23]. From a comprehensive review of the state of the art, the authors discovered that there is currently a lack of information on procedures that work to minimize concentration polarization due to increases in feedwater temperature to an RO process experimentally. For this reason, the present research study focuses on the evaluation of concentration polarization in RO membranes by varying the feed water temperature under different TDS concentrations in order to increase product water quality and reduce the energy consumption of the process.

## 2. Materials and Methods

**Figure 2:**
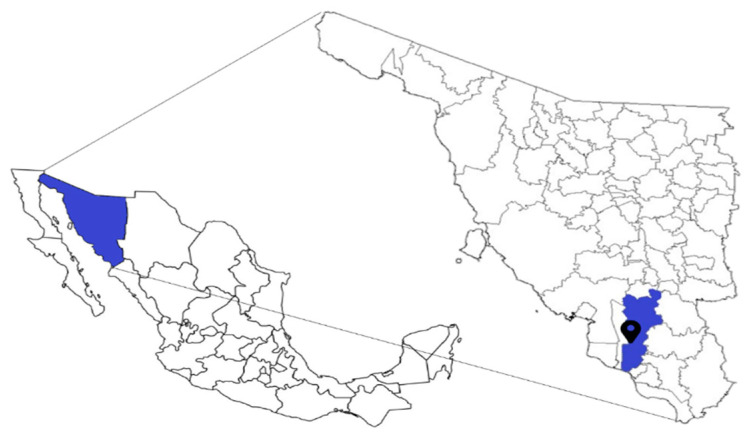
Location of the study area.

Experiments on the effect of varying feed water temperature (23, 25.5, 28, and 35 °C) and inlet water concentration (5000 and 10,000 mg L^−1^) on the concentration polarization in the RO membranes were conducted at the Marine and Brackish Water Desalination Laboratory using Renewable Energies located at the Instituto Tecnológico de Sonora campus Náinari in Ciudad Obregón, Sonora with coordinates of 27°29’11.11” N and 109°56’26.99” W (Figure 2).

The delimitations of the present experimental study are established according to the capacity of the RO water desalination plant, which is 10 L min^−1^ and has a 260 L storage tank. Working with a recovery rate of 50%, the experimental tests have a maximum duration of 20 min. Another conditioning factor is that RO membranes have a maximum temperature resistance between 40 and 45 °C since these temperatures promote the proliferation of biofouling. Therefore, it is proposed to work with temperatures of 23, 25.5, 28, and 35 °C. Two electrical heating elements of 1100 W each were used to heat the water. Infrared equipment was used to measure the temperature during the whole experiment. The residence time throughout the reverse osmosis process is 20–24 min. In order to control the temperature of the water inside the feed tank that will enter the reverse osmosis process, a stirrer was used for 2 min to homogenize the total mixture of the 260 L of water in the feed tank. If the temperature is within the different required ranges of 23, 25.5, 28, and 35 °C, the test is started according to the established design of experiments. A tolerance of ±0.5 °C was considered for the tests and for the temperature setting considered. For the preparation of each sample, the 260 L feed tank was filled with water from the city network of approximately 270 mg L^−1^, and the temperature was measured using a Fluke Model 62 Mini infrared thermometer; in cases where it was necessary to lower the water temperature, cooling systems were used to bring it to the desired temperature.

Subsequently, the electrical conductivity was measured with YSI Model 556 multiparameter equipment and by means of Equation (1).

(1) TDS=EC*644

where *TDS* is the concentration of the feed water (mg L^−1^) and *EC* is the electrical conductivity (mS cm^−1^).

Once the feed water was characterized, the corresponding amount of synthetic sea salt (mg) of the Reef Crystals Reef Salt type manufactured by Instant Ocean, MN, USA, was added, which was calculated using Equation (2). Subsequently, the solution was mixed by stirring for approximately 5 min.

(2) $$Salt=C_{2}V_{1}-C_{1}V_{1}$$

where $C_{1}$ is the concentration of the city network water (mg L^−1^), *V*_1_ is the volume of the feed tank in (L), and $C_{2}$ is the desired concentration (5000 mg L^−1^ or 10,000 mg L^−1^).

In order to protect the RO membranes and ensure the reliability of the experiment, chemical washes were performed with 50 g of Avista Technologics RoClean P112 cleaner, San Marcos CA, USA, diluted in 260 L of city network water, representing a 19.2% concentration. This was followed by a wash with Avista brand Vitec 4000 membrane antifouling agent, San Marcos CA, USA. One liter of solution was prepared where 300 mL corresponded to the chemical while the remaining 700 mL were distilled water, representing a 30% concentration. The liter of antiscalant prepared was supplied to a tank of 3.78 L in total capacity so that the Milton Roy, Pasadena, CA, USA dosing pump injected the solution to the feed water in a combination of 40% stroke and 40% pulsation, in such a way that it promoted the dragging of salts and biofouling out of the system through the reject water.

### 2.1. Description of the Reverse Osmosis System

**Figure 3:**
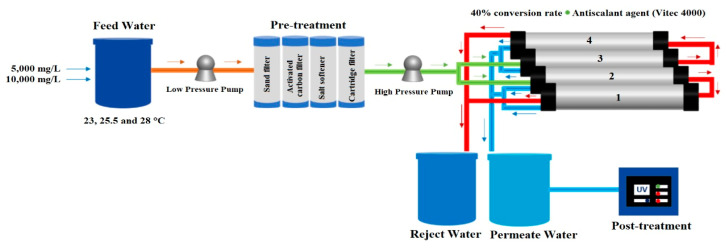

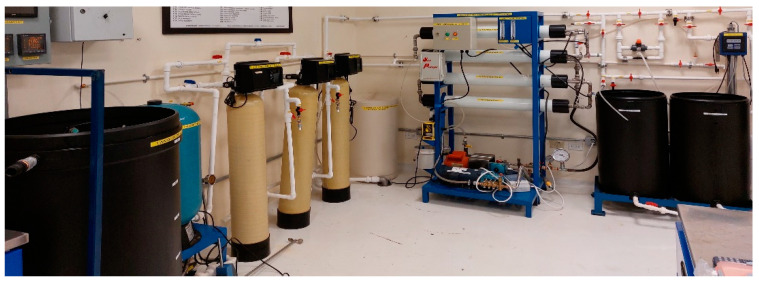
Reverse osmosis desalination system. Above schematic; below real [24].

A 10 L min^−1^ RO desalination plant was used, consisting of three stages: physical and chemical pretreatment, treatment, and post-treatment. The design is shown in Figure 3. The feed water circulated through the three pretreatment filters using an ESPA model 2025SL low-pressure pump. The first filter to pass through was the sand filter, which retained particles larger than 50 µm, followed by the activated carbon filter, which removed the chlorine and organic matter present in the water and removed the odor and color of the water. Both the sand filter and the activated carbon filter are Pentair Structural Poly Glass 8”×44” filters with a capacity of 2.12 m^3^ each. Finally, the water was passed to the Aquor PP Sedimenter Filter 20” 5 µm SFCPP-205 cartridge filter.

A Cat Pumps Model 231 high-pressure pump was used to convey the water to the treatment stage of the reverse osmosis plant. Four Hydranautics SWC4 Max 8”×40” spiral wound composite polyamide membranes placed in a frame were used for the experiment. These seawater membranes have an average salt rejection rate of 99.8%, withstand a maximum pressure of 8273.7 kPa, and a maximum temperature of 45 °C, the active area of each membrane is 40.8 m^2^, and a permeate flux of 27.1 m^3^ d^−1^. The membranes are classified as 1, 2, 3, and 4 from the lower to the upper membrane. Membranes 2–3 are in parallel with each other; however, 1–2 and 3–4 are arranged in series, as shown in Figure 3.

During the process, Vitec 4000 antiscalant was injected into the system before passing through the high-pressure pump to reach the RO membranes. The permeate and reject volumetric flow rates (L min^−1^) were measured by rotameters. The volumetric flow rate ratio allowed the calculation of the recovery percentage by means of Equation (3). In addition, the permeate concentration and salt rejection were calculated using Equations (4) and (5). In the last stage of the process, the permeate water that came out of the rotameter was directed through the pipes to the Wedeco UV Technologies Inc. ultraviolet light lamp NLR1845WS, New York, NY, USA, and then stored in the permeate tank (100 L).

The percentage of recovery (%*R*) is determined with:

(3) $$\%R=\frac{Q_{p}}{Q_{f}}*100$$

where $Q_{p}$ is the permeate flow rate (m^3^ s^−1^) and $Q_{f}$ is the feed flow rate (m^3^ s^−1^)

The permeate concentration (*C_P_*) and salt rejection (*R_S_*) were determined with:

(4) $$C_{p}=\left(1-R_{s}\right)*\left(\frac{C_{f}+C_{R}}{2}\right)$$

(5) Rs=Cf−CpCf

where $C_{p}$ is the permeate concentration (mg L^−1^), *R_S_* is the salt rejection (%), $C_{f}$ is the feed concentration (mg L^−1^) and $C_{R}$ is the rejection concentration (mg L^−1^).

### 2.2. Experiment Description

The experiment consists of 6 combinations with 3 replicates each in order to analyze the effect of concentration polarization (β) at different temperatures and concentrations in the feed water. Therefore, a total of 18 experimental tests were carried out. The 18 experimental tests were carried out at a conversion percentage of 50% so that for every 10 L min^−1^ of feed water, 5 L min^−1^ of permeate water and 5 L min^−1^ of reject water were obtained.

### 2.3. Experiment Description

For the analysis of the concentration polarization (β), data were measured on the pressures of the low- and high-pressure pumps (kPa), volumetric flow rates (L min^−1^), temperatures (°C), and densities (kg m^−3^) of the feed, permeate and reject water, as well as their concentrations (mg L^−1^) at different stages of the RO desalination process. From the measured data and using Equations (6)–(11), inherent variables were calculated, such as the mass transfer coefficient and diffusivity, which were used for the determination of the concentration polarization and its analysis.

The mass transfer coefficient (*kmt*) is determined with:

(6) $$k_{mt}=\frac{Sh*D_{f}}{d_{h}}=\frac{0.023Re^{0.8}Sc^{1/3}D_{f}}{d_{h}}$$

where $k_{mt}$ is the mass transfer coefficient (m s^−1^), Sh is the sherwood number (-), $D_{f}$ is the feed diffusion, $d_{h}$ is the hydraulic diameter, Re is the Reynolds number (-) and Sc is the Schmith number (-).

The diffusivity of the feed water ($D_{f}$) is determined with:

(7) $$D_{f}=\frac{\left(1.173*10^{-15}\right)*T_{p}*{\left(2.26*58.44\right)}^{-0.5}}{0.02^{0.6}*\mu_{p}}$$

where $T_{p}$ is the temperature of the permeate water (°C) and $\mu_{P}\;$ is the viscosity of the permeate water (Pa s).

The permeate flux rate (*J_P_*) is determined with:

(8) $$J_{P}=\frac{Q_{P}}{A_{m}}$$

where $J_{p}$ is the permeate flux (m s^−1^), $Q_{p}$ is the permeate flow rate (m^3^ s^−1^) and $A_{m}$ is the membrane area (m^2^).

The Sherwood [25,26] and Reynolds number were determined with:

(9) $$Sh=\frac{k_{mt}d_{h}}{D_{f}}=0.023Re^{0.8}Sc^{1/3}$$

(10) $$Re=\frac{\rho \;v\;dh}{\mu p}$$

where ρ is the permeate density (kg m^−3^), v is the water velocity (m s^−1^)

The Schmith number and concentration polarization are determined with:

(11) $$Sc=\frac{\mu_{f}}{\rho \;D_{f}}$$

(12) $$\beta =\exp\left(\frac{J_{p}}{k_{mt}}\right)$$

where $\mu_{P}$ is the feed water viscosity (Pa s) and β is the concentration polarization (-)

The temperature correction factor *(TCF_P_)* for permeate ($TCF_{P}$*)* and solute ($TCF_{S}$*)* are determined by the following equations [27]:

(13) $$TCF_{P}=\exp\left[0.0343\;\left(T-25\right)\right]\;\;\;\;\;\;\;<25\;{}^{\circ}C$$

(14) $$TCF_{P}=\exp\left[0.0307\;\left(T-25\right)\right]\;\;\;\;\;\;\;>25\;{}^{\circ}C$$

(15) $$TCF_{s}=1+0.05\;\left(T-25\right)\;\;\;\;\;\;\;\;\;\;\;\;\;\;<25\;{}^{\circ}C$$

(16) $$TCF_{s}=1+0.08\;\left(T-25\right)\;\;\;\;\;\;\;\;\;\;\;\;\;\;>25\;{}^{\circ}C$$

Temperature affects both the osmotic pressure and the permeability of water across the membrane [28].

### 2.4. Initial Investment and Permeate Water Costs

Using DesalData 2021 data [29], the initial investment costs in brackish water reverse osmosis (BWRO) plants and seawater reverse osmosis (SWRO) plants for Mexico have been estimated. The relationship between the investment cost ($/USD) and the installed capacity (m^3^ d^−1^) of desalination plants using brackish and seawater as input water to the process will be established. This will be of great help to decision makers since it will be possible to estimate the initial investment cost or size of the current or future plant to be installed.

## 3. Results

### 3.1. Feed Water Characterization

The physicochemical parameters of the feedwater from the 5000 and 10,000 mg L^−1^ tests prepared in the laboratory are shown in Table 1.

### 3.2. Permeate Concentration and Salt Removal

**Figure 4:**
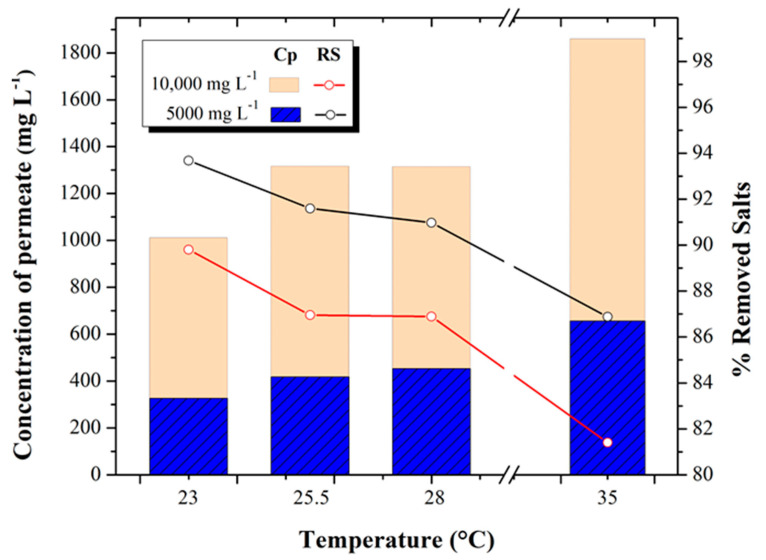
Permeate concentration (mg L^−1^) and percent salt removal at different temperatures and concentrations.

Figure 4 shows the relationship between the behavior of permeate salinity and percent salt removal of the RO process with feed water at different temperatures (23, 25.5, 28, and 35 °C) and concentrations (5000 and 10,000 mg L^−1^ TDS).

In the 5000 mg L^−1^ test, an increase in the permeate salt concentration was obtained from 326.15 ± 36.21 mg L^−1^ to 656.03 ± 41.79 mg L^−1^, which represents an increase of 50% for the temperature increase from 23 to 35 °C tests, respectively. On the other hand, in the 10,000 mg L^−1^ test, the permeate concentration reached values of 1011.08 ± 294.43 mg L^−1^, 1316.33 ± 295.43 mg L^−1^, 1314.18 ± 250.03 mg L^−1^, and 1859.13 ± 81.27 mg L^−1^ in the 23, 25.5, 28 and 35 °C tests, respectively, equivalent to an average increase of 44%. With respect to the percentage of salt removal for the 5000 mg L^−1^ tests, it decreased from 93.68% to 86.88%, while in the 10,000 mg L^−1^ tests, it decreased from 89.8 to 81.40% for the temperature increase from 23 to 35 °C tests, respectively.

Tests performed at different concentrations (5000 and 10,000 mg L^−1^) have similar trends. In both cases, the permeate concentration increases while the percentage of salt removal decreases as the temperature increases. This occurs because the components that make up the dense surface of the membrane -*polyamide*- expand when exposed to temperature increases so that the salts in the feed water dissolve in greater proportion and pass through the membrane more easily, causing a higher concentration of salts in the permeate stream -*salt passage*-. These behaviors coincide with those reported by Elsayed [30], which show that, at a higher temperature, there is higher salinity in the permeate and lower salt removal. That is why a temperature correction factor is applied. It is usually accepted that the pressure and permeate flow rate increase by about 0.5 bar and 3% for each 1 °C increase in temperature, respectively. 

### 3.3. Permeate Concentration and Mass Transfer Coefficient

**Figure 5:**
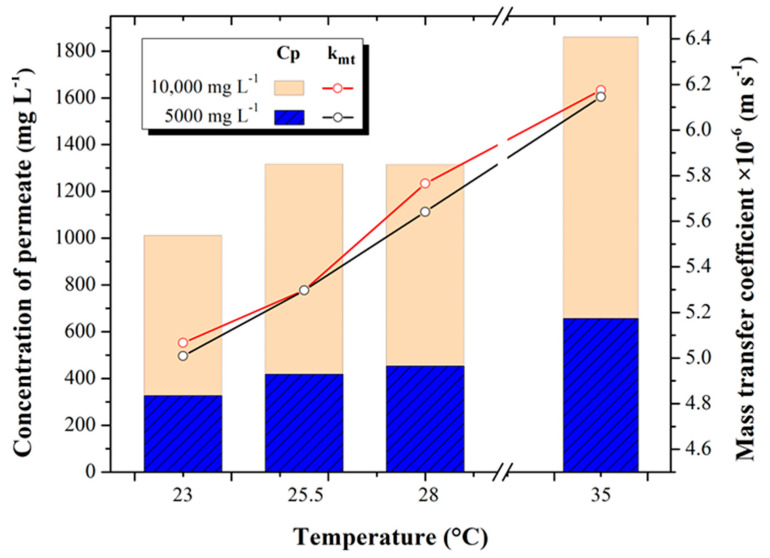
Permeate concentration (mg L^−1^) and mass transfer coefficient (m s^−1^) at different concentrations and temperatures.

Figure 5 shows the behavior of the salinity obtained in the permeate with respect to the mass transfer coefficient calculated with the data measured in the experimental desalination tests for the 5000 and 10,000 mg L^−1^ tests at different temperature increments 23, 25.5, 28, and 35 °C.

Permeate salinity concentration increased from 326.15 ± 36.21 to 656.03 ± 41.79 mg L^−1^ in the 5000 mg L^−1^ tests, while minimum concentrations of 1011.08 ± 296.24 and maximum concentrations of 1859.33 ± 52.30 mg L^−1^ were recorded in the 10,000 mg L^−1^ tests, for the temperature increase from 23 to 35 °C tests, respectively for both concentrations.

The minimum and maximum results obtained for the mass transfer coefficient are 5.016 × 10^−6^ ± 4.07 × 10^−8^ and 6.14 × 10^−6^ ± 5.54 × 10^−8^ m s^−1^ for the 23 and 35 °C tests at 5000 mg L^−1^, respectively. For the 10,000 mg L^−1^ tests, the mass transfer coefficient increased from 5.056 × 10^−6^ ± 6.93 × 10^−8^ m s^−1^ (at 23 °C) to 6.174 × 10^−6^ ± 1.16 × 10^−7^ m s^−1^ (at 35 °C), increasing by 11.87 and 13.8%, respectively.

The behaviors observed above are supported by the results of Joseph [31], who, with brackish and seawater, evaluated the behavior of different variables when working with different temperatures in a RO process; he reports that increasing the concentration and temperature of the feed water also increases both the concentration of the permeate and its diffusivity, the latter directly affecting the mass transfer coefficient.

The results show that for brackish water, it is possible to increase the feed water temperature and favor mass transfer for the three temperatures studied. However, in the ranges from 25.5 °C to 35 °C at a concentration of 10,000 mg L^−1^, the final concentration of the permeate is observed to be above 1000 mg L^−1^, which means that the permeate water is not suitable for consumption, according to Mexican regulation NOM-127-SSA1-2021 [32]. Therefore, a very important finding is that for concentrations higher than 10,000 mg L^−1^, it is not advisable to increase the temperature since the increase in the mass transfer coefficient will increase the passage of salts, causing the permeate to exceed the recommended limits. In the case of the experiment presented by Kim [33], when evaluating the behavior of seawater by increasing the temperature in an RO process, he observed that the permeate water quality decreased significantly.

### 3.4. Operating Pressure and Specific Energy Consumption

**Figure 6:**
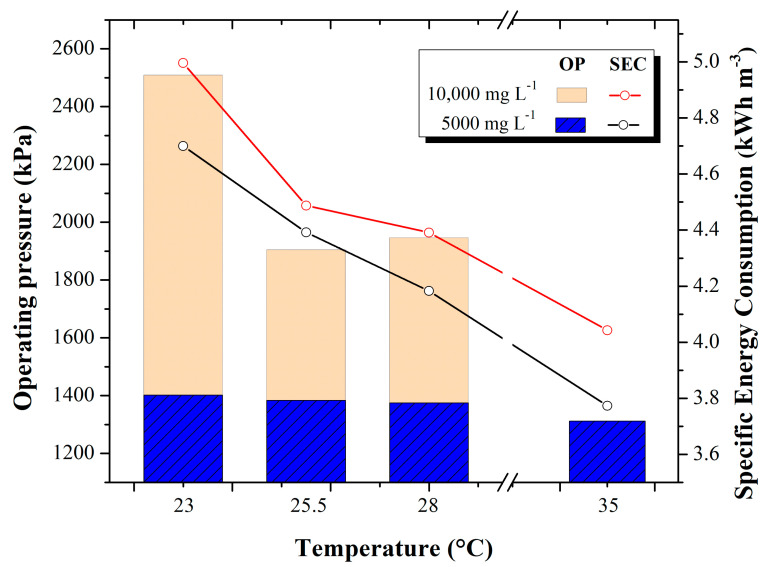
Behavior of operating pressure (kPa) and specific energy consumption (kWh m^−3^) at different temperatures and concentrations.

The relationship between the operating pressure used for the desalination tests and the specific energy consumption is shown in Figure 6.

In the 5000 mg L^−1^ tests, an operating pressure of 1379 kPa was maintained. Energy consumption decreased as feedwater temperature increased. Values in specific energy consumption range from 4.7 ± 0.46 kWh m^−3^ (at 23 °C) to 3.773 ± 0.109 kWh m^−3^ (at 35 °C). This increase in temperature represents a decrease in specific energy consumption of 0.590 kWh m^−3^. On the other hand, at a concentration of 10,000 mg L^−1^, the changes in operating pressure are greater. That is, at 23 °C feedwater temperatures, the required pressure was 2503 kPa on average, while at 35 °C temperatures, lower pressure was required, i.e., 1944.3 kPa on average. Specific energy consumption values range from 4.99 ± 0.22 kWh m^−3^ (at 23 °C) to 4.042 ± 2.9 × 10^−15^ kWh m^−3^ (at 35 °C). This temperature increase represents a decrease in specific energy consumption of 0.72 kWh m^−3^.

It is important to highlight that a large-scale RO plant at ambient temperature consumes on average from 3 kWh m^−3^ to 3.8 kWh m^−3^; in this sense, for this study, a decrease of 0.590 kWh m^−3^ and 0.72 kWh m^−3^ was achieved at 5000 and 10,000 mg L^−1^, respectively, which enhances the effect of increasing temperature in decreasing specific energy consumption, certainly this finding can be applied on a large scale in the industrial sector, [34].

Although the specific power consumption and the operating pressure decrease with increasing temperature, this is something that the decision maker will have to face since, as discussed in Section 3.3, increasing temperature increases the concentration of water permeated, which is controversial since one of the purposes of the desalination process is to obtain quality water. In this way, a balance point must be found regarding what temperature should be fed to the process that reflects a decrease in energy consumption without jeopardizing the quality of the permeated water. In addition, it is necessary to know this relationship, as there will be an unnecessary expense of the time and energy necessary to heat the feed water.

As the temperature of the feed water increases, it is easier for the water to pass through the RO membrane because the salts are mostly dissolved, and those that are retained by the membrane are carried away with greater fluidity through the rejection flow, avoiding clogging of the membrane and a decrease in the pressure required by the pump to make reverse osmosis. Koutsou [35] conducted research evaluating the behavior of brackish and seawater at different temperatures in a RO process and reports that the physicochemical parameters of the water are directly influenced by the variation of temperature and salinity of the water; however, he did not study the relationship of pressure and energy consumption that is presented in this work. He mentions that when working with brackish and saline waters, optimal results are obtained in terms of the benefit of specific energy consumption. On the other hand, when working with higher concentrations, the same tendency as the 10,000 mg L^−1^ tests is presented, where at the beginning, as the temperature increases, the specific energy consumption decreases.

### 3.5. Diffusivity and Concentration Polarization

**Figure 7:**
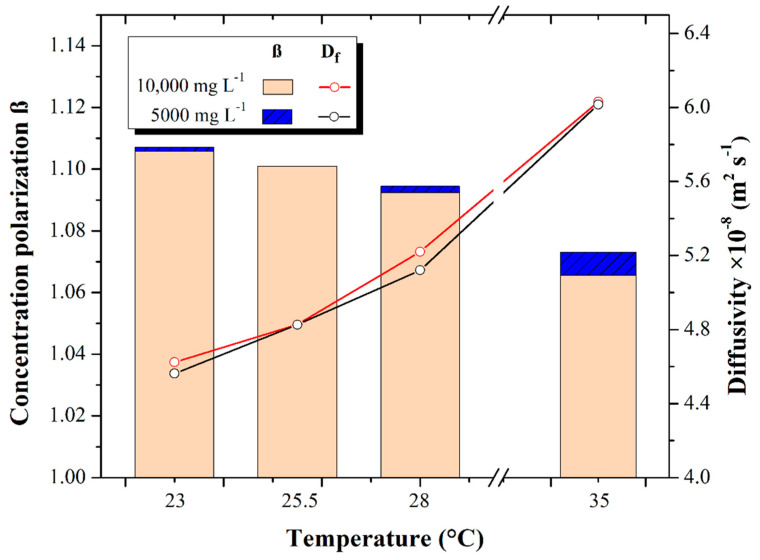
Feedwater diffusivity (m^2^ s^−1^) and concentration polarization β at different concentrations and temperatures.

The behaviors of the concentration polarization factor (β) and feedwater diffusivity at different temperatures (23, 25.5, 28, and 35 °C) and concentrations (5000 and 10,000 mg L^−1^ TDS) are shown in Figure 7.

The concentration polarization (β) presented a value of 1.106 ± 0.00096 as a maximum for the 23 °C test and 1.094 ± 0.00097 as a minimum for the 28 °C test, both at 5000 mg L^−1^. For the 10,000 mg L^−1^ tests, the concentration polarization β was found to have a maximum of 1.106 ± 0.0015 when operating at 23 °C and 1.092 ± 0.0019 working with 28 °C feed water temperature. Such values are within the range established by Kucera [10] for the minimization of scaling formation in the membrane, ranging from 1 to 1.2 for the concentration polarization (β). From the above, it is stated that there is a correlation between the increase in feedwater temperature and the decrease in the concentration polarization (β).

The concentration polarization β decreases because the physicochemical properties of water and salts are directly affected by the temperature increase. The viscosity and density of the water decrease while the salt dissolves better in the solution, so the dissolved salts pass through the membrane, and the retained salts flow more easily through the rejection flow. Rodriguez [24] investigated the behavior of the concentration polarization (β) with 5000 mg L^−1^ feed water at different temperature increments and reported a decrease in the concentration polarization (β).

The feed water diffusivity obtained was 4.57 × 10^−8^ ± 5.27 × 10^−10^ m^2^ s^−1^ as a minimum in the 23 °C test at 5000 mg L^−1^ and 6.015 × 10^−8^ ± 6.03 × 10^−10^ m^2^ s^−1^ as maximum for the 35 °C tests at 5000 mg L^−1^, increasing the feed water diffusivity by 24.18%. Similarly, the water diffusivity for the 10,000 mgL^−1^ tests resulted in 4.623 × 10^−8^ ± 5.607 × 10^−10^ m^2^ s^−1^ at its minimum at 23 °C and 6.030 × 10^−8^ ± 2.923 × 10^−10^ m^2^ s^−1^ at its maximum at 28 °C, increasing the diffusivity 23.3%.

The above indicates that the increases in temperature and diffusivity of the feed water are proportional. On the other hand, the concentration polarization decreases. This coincides with the results of Mukesh-Sharma [36], who report that temperature has significant repercussions in the water desalination process and mentions that increasing the temperature results in a reduction in the concentration polarization and in the viscosity of the feed water, which favors better mass transfer and diffusivity through the membrane. 

### 3.6. Investment and Desalinated Water Cost

**Figure 8:**
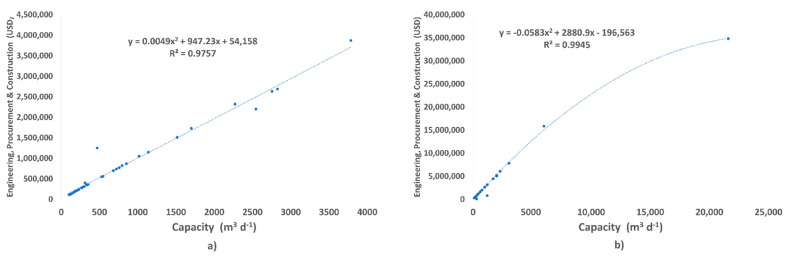
Correlation between initial investment and BWRO (**a**) SWRO (**b**) capacity. Desaldata [29].

Figure 8 shows the quadratic relationship of the cost of 37 reverse osmosis brackish water plants and 39 reverse osmosis seawater plants installed versus the installed capacity m^3^ d^−1^ of desalination plants installed in Mexico. The resulting equations describe an approximation to be had on the initial investment cost or the installed or to-be-bid-built plant capacity in a short time.

Also, Table 2 shows that feedwater temperature has a direct impact on the costs of brackish water and seawater desalination plants. For salinity levels of 5000 and 10,000 mg L^−1^, the temperature is inversely proportional to the different desalination plant costs; therefore, as higher-temperature water is fed, the total plant cost decreases.

Table 2 shows the total cost (TC) in $USD m^−3^ of desalinated water for different temperatures in the brackish feedwater at 23, 25.5 and 35 °C at 5000 mg L^−1^ and 5000 mg L^−1^ with their different costs and percentages. Specific energy consumption (A), maintenance (B), labor (C), management (D), and chemicals (E).

## 4. Conclusions

The present investigation evaluated the concentration polarization (β) and inherent variables in the RO process with feed water at temperatures of 23, 25.5, 28, and 35 °C and at concentrations of 5000 and 10,000 mg L^−1^. Although the effect of temperature on concentration polarization has been studied, it is limited to theoretical studies that consider fixed concentrations for feed water. Therefore, the results of this study allow us to experimentally analyze the effect of feed water temperature for different concentrations (5000 and 10,000 ppm) and to determine whether the decrease in concentration polarization increases or decreases with increasing feed water concentration. 

The increase in temperature from 23 °C to 35 °C in the feed water generates a positive impact on the concentration polarization in the RO membranes. For the 5000 mg L^−1^ tests, a decrease of 3.10% was achieved, while for the 10,000 mg L^−1^ tests, it decreased by 3.64%. All concentration polarization (β) values obtained resulted below the established limit of 1.2 to prevent the formation of salt scaling in the membrane layer. Furthermore, the results obtained for both concentrations indicate that with increasing temperature from 23 to 35 °C in the feed water, both its diffusivity and mass transfer coefficient increase by 31.86 and 22.71%, respectively. These increase in diffusivity and mass transfer coefficient at concentrations of 5000 and 10,000 mg L^−1^ by means of temperature increase (23 to 35 °C) produces an increase in permeate salinity concentration and a decrease in percent salt removal. The temperature of 35 °C causes a greater decrease in the SEC and concentration polarization due to the better diffusion of the solutes in the permeate water, decreasing the presence of fouling in the membrane film.

The specific energy consumption and the operating pressure in the two reverse osmosis desalination tests showed a decrease as the temperature of the feed water increased from 23 to 35 °C. In order not to affect the concentration and quality of the permeated water due to the effect of the increase in temperature in the feed water, it was found that the ideal temperature to work with water of 10,000 mg L^−1^ -*and probably higher*- due to the trend shown is of 25.5 °C. This is due to the fact that the higher the temperature in the feed water, the diffusivity and mass transfer increase. 

The research demonstrates the effect of the variation in feed water temperature in a RO system, benefiting the process by reducing energy consumption and the scaling of salts deposited on the membrane surface, causing a reduction of chemicals applied to the process and, therefore, a decrease in the overall cost of the process. Reducing the concentration polarization will allow advances in the scientific community by developing innovative methodologies to understand, study and perform laboratory tests to reduce them. On the other hand, the industrial sector will apply this scientific knowledge for the reduction of the concentration polarization β. All of the above potentiates the implementation of RO desalination technology as an attractive alternative to reduce current water shortage problems, providing water in quantity and quality to populations located in urban centers and/or remote communities near coastlines.

## Figures and Tables

**Table 1:** Physicochemical characterization of feed water.

Parameters	5000 mg L^−1^	10,000 mg L^−1^
Volume (L)	260.00	260.00
TDS (mg L^−1^)	5013.00	10,003.00
pH	7.80	7.90
Electrical conductivity (mS cm^−1^)	7.78	15.53
Hardness CaCO_3_ (mg L^−1^)	718.20	1436.40
Chlorine (mg L^−1^)	0.00	0.00
Turbidity (NTU)	0.00	0.01

**Table 2:** Permeate water cost.

T (°C)	A (62%)			B (27%)	C (5.2%)	D (3.2%)	E (2.6%)	TC
	kWh m^−3^	USD kWh^−1^	USD m^−3^	USD m^−3^				
Feed Water (5000 mg L^−1^)								
23	4.7	1.04	0.244	0.110	0.021	0.013	0.011	0.399
25.5	4.45	1.04	0.231	0.104	0.020	0.012	0.010	0.378
28	4.18	1.04	0.217	0.098	0.019	0.012	0.009	0.355
35	4.11	1.04	0.214	0.096	0.019	0.011	0.009	0.349
Feed Water (10,000 mg L^−1^)								
23	4.95	1.04	0.257	0.116	0.022	0.014	0.011	0.420
25.5	4.52	1.04	0.235	0.106	0.020	0.013	0.010	0.384
28	4.38	1.04	0.228	0.102	0.020	0.012	0.010	0.372
35	4.23	1.04	0.220	0.099	0.019	0.012	0.010	0.359

## Data Availability

Not applicable.

## Nomenclature

Symbols		
$A_{m}$	Effective membrane area	m^2^
$C_{1}$	Salt concentration of the city network water	mg L^−1^
$C_{2}$	Desired salt concentration	mg L^−1^
D	Solute diffusivity	m^2^ s^−1^
$d_{h}$	Hydraulic diameter	m
EC	Electrical conductivity	mS cm^−1^
$J_{p}$	Volumetric permeate flux	m s^−1^
$k_{mt}$	Mass transfer coefficient	m s^−1^
OP	Operating pressure	kPa
Q	Volumetric flow rate	L min^−1^
Re	Reynolds number	-
Sh	Sherwood number	-
Sc	Schmidt number	-
SEC	Specific energy consumption	kWh m^−3^
T	Temperature	°C
TDS	Total dissolved solids	mg L^−1^
v	Water velocity in membrane module	m s^−1^
$V_{1}$	Volume of the feed tank	L
β	Polarization factor	-
μ	Fluid viscosity	Pa s
ρ	Density of water	kg m^−3^
Subscripts and abbreviations		
ED	Electrodialysis	
RO	Reverse osmosis	
p	permeate	
f	feed	
R	rejection	
m	membrane	
